# Whole exome sequencing of pediatric leukemia reveals a novel InDel within FLT-3 gene in AML patient from Mizo tribal population, Northeast India

**DOI:** 10.1186/s12863-022-01037-x

**Published:** 2022-03-28

**Authors:** Andrew Vanlallawma, Doris Lallawmzuali, Jeremy L. Pautu, Vinod Scaria, Sridhar Sivasubbu, Nachimuthu Senthil Kumar

**Affiliations:** 1 Department of Biotechnology, Mizoram University, Aizawl, Mizoram 796004 India; 2 Department of Pathology, Mizoram State Cancer Institute, Zemabawk, Aizawl, Mizoram 796017 India; 3 Department of Medical Oncology, Mizoram State Cancer Institute, Zemabawk, Aizawl, Mizoram 796017 India; 4 CSIR - Institute of Genomics and Integrative Biology, South Campus, Mathura Road, New Delhi, 110025 India

**Keywords:** Pediatric leukemia, Exome sequencing, FLT3, PTPN11, Non-synonymous, Mizoram

## Abstract

**Background:**

Leukemia is the most common type of cancer in pediatrics. Genomic mutations contribute towards the molecular mechanism of disease progression and also helps in diagnosis and prognosis. This is the first scientific mutational exploration in whole exome of pediatric leukemia patients from a cancer prone endogamous Mizo tribal population, Northeast India.

**Result:**

Three non-synonymous exonic variants in *NOTCH1* (p.V1699E), *MUTYH* (p.G143E) and *PTPN11* (p.S502P) were found to be pathogenic. A novel in-frame insertion-deletion within the juxtamembrane domain of FLT3 (p.Tyr589_Tyr591delinsTrpAlaGlyAsp) was also observed.

**Conclusion:**

These unique variants could have a potential mutational significance and these could be candidate genes in elucidating the possibility of predisposition to cancers within the population. This study merits further investigation for its role in diagnosis and prognosis and also suggests the need for population wide screening to identify unique mutations that might play a key role towards precision medicine.

**Supplementary Information:**

The online version contains supplementary material available at 10.1186/s12863-022-01037-x.

## Background

Leukemia is the most common type of childhood cancer and the incidence is estimated to be 3.1 per 100,000 cases worldwide [1]. Leukemia can be broadly classified according to the type of hematopoietic lineage that turns cancerous as lymphoid or myeloid leukemia and by the progressiveness of the disease as acute or chronic. Previously, the causal root factor for leukemia was thought to be chromosomal translocation [2], however, there are reports that indicate that this translocation alone is not adequate for leukemiogenesis and are even observed during pregnancy [2–4]. Moreover, the translocation does not define the progressiveness of ALL patients [5, 6].

Apart from the chromosomal translocation, studies on nuclear mutational pattern revealed a crucial event in the Acute Myeloid Leukemia (AML) pathogenesis and its clinical significance [7, 8]. The two-hit model of leukemiogenesis captures the key events in the genomic alteration, where the two classes of mutations: one in the genes responsible for growth or survival and the other in the genes responsible for differentiation leading to self-renewability were proposed for leukemiogenesis [9]. Identifying a specific gene mutation in leukemia plays a vital role in its diagnosis, prognosis and also in predicting the disease-free survival rate and recurrence [10].

Next Generation Sequencing (NGS) approach such as Whole Exome Sequencing (WES) has been used in identifying the mutational profiles of different cancers and its subtypes. The mutational profiles of pediatric leukemia have also been studied in different ethnic groups revealing recurrent mutational hotspots, driver genes and variants involved in different pathways: RTK/RAS signaling and its downstream MAPK/ERK signaling, PI3K/AKT and MTOR, JAK/STAT signaling, Notch signaling, WNT/β-catenin, CXCL12, NF-κB, Metabolic and other pathways, including p53 [11–14]. The class of genes that are frequently mutated includes lymphoid/myeloid differentiation, transcription factors, epigenetic regulators, signal transduction, apoptotic regulators [15, 16]. *FLT-3* variants within a particular hotspot region have been reported to be different across different ethnic groups and various types of indels and internal tandem duplication have also been reported [17]. Hence, it is very much essential to study unexplored ethnic groups with high incidences of cancers.

Here, we report whole exome sequencing of pediatric leukemic patients as the first scientific report from Mizo endogamous tribal population, Northeast India wherein the state has the highest incidences of various Cancers in the country [18]. We hypothesize that the high incidence of cancer rate in the population might be a result of unique mutations that are present within the coding regions of the genome. To understand the germline mutations in the population as well as to capture the variants that may be directly responsible for the disease, the present study is a pilot approach to explore the pediatric patient samples.

## Results

Whole exome analysis of pediatric leukemia patients identified 46 non-synonymous exonic variants with allele frequency ≤ 0.05, out of which 16 variants have been reported in ClinVar (Table 1). However, only *MUTYH* variant (p.G143E; dbSNP id: rs730881833) present in AML-M1 patient was reported as likely pathogenic for MUTYH associated Polyposis and Hereditary Cancer Predisposition Syndrome in ClinVar. Non-synonymous exonic gene variants that are not present in ClinVar are listed in Table 2**.** *NOTCH1* variant (p.V1699E) in one patient (AML-M1) was not reported in any database and predicted as pathogenic by 7 different prediction tools using VarSome [19]. *PTPN11* variant (p.S502P) present in one patient (AML-M1) was identified which was also not present in ClinVar. Sanger Validation of point mutation observed in this study are shown in Supplementary Figs. 1, 2 and 3.

**Table 1:** Non-synonymous exonic variants that matched with ClinVar with their clinical significance and disease associated

Chr	Pos	Ref	Alt	Gene	Clinical Significance from ClinVar	Disease associated
11	108,098,555	A	G	ATM	Conflicting interpretations of Pathogenicity	Ataxia-telangiectasia syndrome, Hereditary cancer-predisposing syndrome
11	108,159,732	C	T	ATM	Benign / Likely Benign	Ataxia-telangiectasia syndrome, Hereditary cancer-predisposing syndrome
11	119,156,193	C	T	CBL	Benign / Likely Benign	Rasopathy, Noonan-Like Syndrome Disorder
12	49,434,409	G	A	KMT2D	Benign	Kabuki syndrome
1	45,797,401	G	A	MUTYH	Conflicting interpretations of Pathogenicity	MYH-associated polypopsis, Hereditary cancer-predisposing syndrome
**1**	**45,797,914**	**C**	**T**	**MUTYH**	**Pathogenic / Likely Pathogenic**	**MYH-associated polypopsis, Hereditary cancer-predisposing syndrome**
1	45,800,146	C	T	MUTYH	Benign, Uncertain Significance	MYH-associated polypopsis, Hereditary cancer-predisposing syndrome
1	45,800,167	G	A	MUTYH	Benign, Uncertain Significance	MYH-associated polypopsis, Hereditary cancer-predisposing syndrome
18	42,643,270	G	T	SETBP1	likely Benign	Schinzel-Giedion syndrome
1	85,742,023	C	A	BCL10	Benign	Immunodeficiency 37
20	31,022,469	G	A	ASXL1	Benign	C-like syndrome
22	23,654,017	G	A	BCR	Uncertain Significance	ALL and AML
4	106,158,550	G	T	TET2	Not provided	
4	55,589,830	A	G	KIT	Uncertain Significance	Gastrointestinal stroma tumor
9	139,401,375	C	T	NOTCH1	Uncertain Significance	Adams-Oliver syndrome 5, Cardiovascular phenotype
9	139,410,139	T	C	NOTCH1	Uncertain Significance	Adams-Oliver syndrome 5

*Chr* Chromosome Number, *Pos* Position, *Ref* Reference Allele, *Alt* Alternate Allele

**Table 2:** Non-synonymous exonic variants not matched in CIViC and ClinVar with their OMIM phenotype and pathogenicity prediction

Sample	Gene	Ref	Counts (%)	Alt	Counts (%)	Total reads	AA change	Hom /Het	OMIM phenotype and Mode of inheritance	S_P	P_P	MT_P
GDN4252	BCL10	C	49 (43%)	A	64 (56%)	114	A5S	Het	Male germ cell tumor, somatic	T	B	N
GDN4253	BCL10	C	28 (55%)	A	23 (45%)	51	A5S	Het	Male germ cell tumor, somatic	T	B	N
	BIRC3	A	28 (52%)	G	26 (45%)	54	K260R	Het	–	T	B	N
	NOTCH1	T	16 (38%)	C	26 (62%)	42	I567V	Het	–	T	B	D
	ATM	G	112 (53%)	T	99 (47%)	211	C1482F	Het	T-cell prolymphocytic leukemia, somatic	T	B	N
GDN4255	BCL10	C	58 (48%)	A	62 (51%)	121	A5S	Het	Male germ cell tumor, somatic	T	B	N
	**NOTCH1**	**A**	**104 (75%)**	**T**	**35 (25%)**	**139**	**V1699E**	**Het**	**–**	**D**	**D**	**D**
	BIRC3	A	57 (55%)	G	46 (45%)	103	K260R	Het	–	T	B	N
	ASXL1	G	90 (49%)	A	95 (51%)	185	D1163N	Het	Myelodysplastic syndrome, somatic	T	B	N
GDN4256	BIRC3	A	14 (39%)	G	22 (61%)	36	K260R	Het	–	T	B	N
GDN4258	MUTYH	G	35 (49%)	A	37 (51%)	72	A230V	Het	–	T	P	D
	KIT	A	47 (44%)	G	59 (56%)	106	I438V	Het	Germ cell tumors, somatic, Leukemia, acute myeloid (Smu,AD)	T	B	D
	ATM	A	68 (46%)	G	80 (54%)	149	H24R	Het	T-cell prolymphocytic leukemia, somatic	T	B	N
	ATM	C	49 (49%)	T	50 (51%)	99	H1380Y	Het	T-cell prolymphocytic leukemia, somatic	T	B	N
	SETBP1	G	14 (47%)	T	16 (53%)	30	E1466D	Het	–	T	B	N
GDN4259	NOTCH 1	C	72 (43%)	T	94 (56%)	167	V1232M	Het	–	T	P	N
	ASXL1	C	51 (49%)	T	53 (51%)	104	Q757X	Het	Myelodysplastic syndrome, somatic	T	0	D
GDN4260	MUTYH	C	66 (55%)	T	54 (45%)	121	G25D	Het	–	T	P	N
	MUTYH	G	55 (51%)	A	52 (49%)	107	P18L	Het	–	T	B	D
	BCL10	C	0 (0%)	A	57 (97%)	59	A5S	Hom	Male germ cell tumor, somatic	T	B	N
GDN4261	**PTPN11**	**T**	**156 (83%)**	**C**	**32 (17%)**	**188**	**S502P**	**Het**	**Leukemia, juvenile myelomonocytic, somatic**	**T**	**P**	**D**
	**FLT3**		**11 (12%)**	**del and ins**	**71 (78%)**	**85**	**YFY589-91delWAGDins**	**Het**	**ALL, AML**	**0**	**0**	**0**
	BCR	–	44 (72)	CCGGins	17 (27)	61	S1092fs	Het	ALL, CML somatic	0	0	0
GDN4262	ATM	A	107 (54%)	C	91 (46%)	198	T1697P	Het	T-cell prolymphocytic leukemia, somatic	T	B	N

*Ref* Reference Allele, *Alt* Alternate Allele, *Counts* Read Counts, *AA Change* Amino acid Change, *Hom/Het* Homozygous/Heterozygous, *S_P *SIFT_Prediction, *P_P* PolyPhen2 Prediction and *MT_P* Mutation taster Prediction. B – Benign, D – Damaging, P – Probably Damaging, N – Neutral, T- Tolerated, 0 – No prediction

## Identification of novel FLT3 InDel in PTPN11 mutation positive patient

**Fig. 1:**
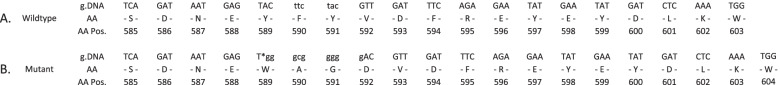
Novel InDel in *FLT-3* identified in AML-M1. **A** Wildtype *FLT-3* (exon 14) depicting the genomic DNA with amino acid it encodes and the position. Bases in lower script indicates the deleted bases (ttctac) in the Mutant type. **B** Mutant *FLT-3* depicting the genomic DNA with amino acid it encodes and the position. * Indicates the position of insertion and bases in lower script (gggcggggg) are the inserted bases

Our study observed two tyrosine amino acid (in 589, 591 position) and phenylalanine (590 position) to be deleted and an in-frame insertion consistent with ITD region [17], four amino acids are inserted [tryptophan (W), alanine (A), glycine (G), aspartic acid (D)- (p.Tyr589_Tyr591delinsTrpAlaGlyAsp)] (Fig. 1). along with *PTPN11* p.S502P from the same patient. NGS based evidence of the indel and its Sanger validation is given in Supplementary Figures (Supplementary Figs. 4 and 5).

## Discussion

Whole exome analysis performed in the germline genomic mutational screening in pediatric leukemia patients showed important heterozygous variants and not in the corresponding mother samples suggesting that it could be a de novo germline mutation or is inherited from the father. The exception was for two homozygous variants, *BCL10*: p.A5S and *ASXL*: p.G652 which were reported as benign in ClinVar for immunodeficiency syndrome and C-like syndrome, respectively. Unreported variants were observed in this study which could be population specific variant.

*MUTYH* encodes an enzyme DNA glycosylase that functions in base excision repair when there is DNA damage from oxidation. *MUYTH* variants are also found in different types of cancers like gastric cancers [20], pediatric high grade midline gliomas patients [21] and in pediatric leukemia [22, 23]. However, a previously unreported variant G143E was found in a two years old girl with AML-M1 subtype with a family history of gastric cancer, but the mother did not carry the same mutation. Nonetheless, as the variant was predicted as pathogenic by three predicting softwares, as well as categorized as MUTYH Associated Polyposis (MAP) and Hereditary Cancer Predisposing Syndrome in ClinVar, the variant might confer loss of the protein function.

*NOTCH1* encodes a transmembrane receptor protein that is required in the differentiation and maturation process and is activated during early embryo or in hematopoiesis [24, 25] Mutations in the PEST and heterodimer domains within *NOTCH1* are found in 50% of T-cell-ALL patients [26]. Mutations in the gene are likely in ALL patients where its role is poorly understood in myeloid malignancies. This may be because activation of the Notch pathway varies between different cell types [27] Fu et al. [28] first reported the *NOTCH1* mutation and even suggested that *NOTCH1* mutations are rare events in AML patients. Study reported that in vivo activation of *NOTCH1* by its ligands arrest AML growth while inhibition confers proliferation [29]. This suggested that *NOTCH1* plays a role as tumour suppressor in AML, furthermore, a novel pathway that activates *NOTCH1* for inhibiting cell growth was identified [30]. The mutation observed in this study as predicted by the prediction softwares (SIFT, PolyPhen2 and Mutation Taster) was deleterious suggesting that *NOTCH1* p.V1699E mutation might confer loss of function and its ability to suppress tumour might be lost. From the aforementioned studies, inactivation or loss of function aids in cell proliferation suggesting that the patient in this study with AML-M1 subtype might have a proliferative advantage as extensive expression of *NOTCH1* especially in M1 and M0 – AML patients with simultaneous expression of CD7 which is a marker for immaturity was observed that reflects in a poor overall survival rate [31].

FLT3 mutations can be classified into point mutations in the Tyrosine Kinase Domain (TKD) and Internal Tandem Duplications (ITD) in the juxtamembrane domain with each accounting for 5 and 25% of patients with AML, respectively. Both these types of mutations resulted in constitutive activation of the gene where the autoinhibitory mechanism is disrupted in the case of ITD and turns to ligand independent FLT3 thereby promoting cell proliferation. Similarly, point mutations in the TKD are in the activation loop that stabilize the active kinase conformation resulting in constitutive activation of its kinase activity [32]. It was also highlighted that approximately 30% of ITDs insert in the TKD1 and not in the JMD [33]. It was observed that 77 pediatric AML patients out of 630 tested positive for ITD out of which 59 had a single duplication and the rest 18 had 2 or 3 ITD’s [17]. Chow et al. [34] also showed that in 569 consecutive adult AML patients 126 (22.1%) harbored FLT3-ITDs. FLT3 mutations occurred in about 35–45% of AML patients with normal karyotype [35]. Consistently, these FLT3-ITD are in-frame mutations with varying size that ranges from 3 to > 1000 nucleotides [36].

Different types of *FLT3-ITD* within a hotspot region have also been reported [35–37]. The InDel found in this study have not been reported earlier. However, the site of duplication observed in this study is fairly consistent with other duplication site which is in the juxtamembrane domain, amino acid 591–599 [17, 34]. This study identified an insertion deletion mutation, where amino acids YFY (positions 589, 590 and 591) are deleted and 4 amino acids (WAGD) are inserted. Y589 and Y591 were reported to be the STAT5 docking site [38] where it activates and expresses an antiapoptotic protein called BCL-xL [39]. Though *FLT3-ITD* was reported to be a driver mutation in AML patients’ initiation of leukemia by *FLT-3* through STAT pathway might not be the case for this patient. However, evading cell death is not the only property of cancers, as acquiring a proliferative advantage is also one of the natures of cancerous cells as proposed in the “two hit model” [9]. The proliferative advantage could be attained for this patient as the tyrosine residue at position 599 in *FLT-3* is still intact and this residue was reported to be the interacting site of *FLT-3* with *PTPN11*. They also showed that the absence of tyrosine residue (Y > F mutant) showed enhanced Erk activation and acquired proliferation and survival advantages when compared with WT-FLT-3 [40]. This could be a potential pathway for its initiation as hyperactive *PTPN11* deregulates the RAS pathway, thereby contributing to its growth [41, 42]. This indel mutation generates a protein with one amino acid longer than the wild type. Length mutation of *FLT-3 – ITD* either by elongation or shortening of the juxtamembrane domain results in gain-of-function and could transform 32D cells, irrespective of the tyrosine residues [43, 44].

Mutations in *PTPN11* are found commonly in JMML patients without RAS and NF1 mutation and are involved in leukemiogenesis by negative regulation of the RAS pathway by conferring growth advantage [45]. Most of the mutations reported in PTPN11 are within the domain N-terminal src-homology-2 (N-SH2) and protein tyrosine phosphatase (PTP) domain. The change of serine to proline results in the loss of S502 – E76 H-bond that is required for its auto-inhibition and thus acquiring an open conformation exposing the catalytic site leading to an increase by 8-fold turnover value of S502P when compared to wild type *PTPN11* in their basal activity [46]. Consistent with other findings, GND4261 has a mutation in PTP domain (p.S502P) with no RAS mutation but positive for *FLT-3* mutants. PTPN11 mutation was found to be seen more among boys [47], but in the present study, the mutation was found in a girl child. In contrast to adult AML patients, where there is no association observed between the two gene mutations, PTPN11 and FLT-3-ITD [47]. However, the sample size is small to define a true association for this population.

## Conclusion

There are four different amino acid changes in the same position of the *PTPN11* (p.S502A, p.S502T, p.S502P, p.S502L) that are reported in ClinVar. A change from serine to alanine was interpreted as pathogenic with clinical conditions like Rasopathy and Noonan Syndrome [48], a change from serine to threonine was interpreted as pathogenic with clinical conditions like Noonan Syndrome 1and Juvenile Myelomonocytic Leukemia [49] and a change from serine to leucine was interpreted as pathogenic with clinical conditions like Noonan Syndrome 1 and Juvenile Myelomonocytic Leukemia [50]. Even though, a change of serine to proline in the same position was reported in few studies in AML and Myelodysplastic Syndrome (MDS) [51], there is no record of the variant’s pathogenicity in its clinical conditions in ClinVar. However, as the other three changes p.S502A, p.S502T, and p.S502L are interpreted as pathogenic, the chance of p.S502P becoming pathogenic is also greatly increased. Additionally, the amino acid residues that are close by (p.R498W/L, p.R501K, p.G503R/V/A/E, p.M504V, p.Q506P, p.T507K) are also reported for Noonan Syndrome in Human Gene Mutation Database (HGMD) [52] which suggest the functional importance of this region.

The two mutations, *NOTCH1* (p.V1699E), and *FLT-3* (p.Tyr589_Tyr591delinsTrpAlaGlyAsp) observed in this study have not been reported and the frequencies are unknown as well. IndiGenomes is a database that had over 1000 healthy Indian genomes where Mizo tribal population are also included in the study [53]. South Asian Genomes and Exomes (SAGE) database consists of 1213 genomes and exome data sets from South Asians comprising 154 million genetic variants [54]. The variants found in our study were not present in the IndiGenomes and SAGE database suggesting that these variants observed might be a disease specific polymorphism for the region. As the sample size of this study is small, stressing the importance of these variants in the population might not be appropriate. However, these findings could be a potential mutational uniqueness towards the population that merits further investigation.

## Materials and methods

### Sample collection

All pediatric leukemia patients totaling to eleven children between 2 and 16 years (median age = 11, 3 girls and 8 boys) who are diagnosed with leukemia and undergoing treatment at Mizoram State Cancer Institute, Aizawl, Mizoram, Northeast India from January–July 2018 were included in this study (Supplementary Table 1). After obtaining informed consent from the parents, 2 ml of peripheral blood was drawn from the patients. Blood sample was also collected from four mothers who are willing to participate. Peripheral blood was collected in EDTA coated vials and stored in -20 °C for DNA isolation.

### DNA isolation and whole exome sequencing

DNA was isolated from whole blood by using QIAamp DNA Mini Kit (CA, USA) as per the manufacturer’s protocol with some modifications. The quality of isolated DNA was checked using Nanodrop (NanoDrop™ 1000 Spectrophotometer, Thermofisher) at optical density (OD) 260 nm. The purity of the isolated DNA was checked by measuring OD at 260/280 for protein contamination as well as 260/230 for RNA contamination. The quality of the isolated DNA was also checked by 0.8% Agarose Gel Electrophoresis. After the required concentration of 100 ng for library preparation was obtained, DNA library was prepared by using Illumina v4 TruSeq Exome library prep as per the manufacturer’s protocol. The sequencing and data analysis was carried out at CSIR- IGIB, New Delhi.

### WES data analysis

Whole Exome Sequencing was performed using Illumina HiSeq 2500 and generated approximately 52.2 million reads that passed Quality Control (QC) with 52.1 million reads (99.97%) aligned to the reference genome (hg19) per sample (Supplementary Table S2). GATK haplotype caller was used for calling germline variants from the generated BAM files [55]. The VCF file was annotated using ANNOVAR [56].

### Prioritization of variants

**Fig. 2:**
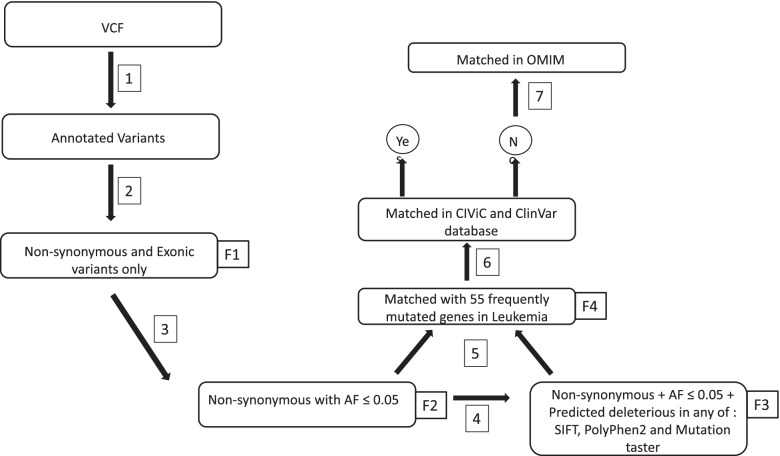
Prioritization of variants for whole exome data. F1 to F4: Filter’s applied. 1: Raw VCF file annotated using ANNOVAR; 2: Selection of non-Synonymous exonic variants from the annotated variants; 3: Selection of variants having allele frequency lower than 0.05; 4: Selection of variants that are predicted as deleterious in any of the two-predicting software (SIFT, PolyPhen2, Mutation Taster); 5: Matching with frequently mutated genes associate with leukemia; 6: Matching with CIViC and ClinVar database; 7: Interpreting using OMIM database

The quality of the raw read fastq files were checked twice before and after trimming the adapter sequence and the low-quality reads by Trimmomatic software [57] and FastQC [58]. Processed fastq files were mapped on human reference genome (hg19) using BWA-MEM [59]. Variant calling was done using GATK haplotype caller [55] and the vcf file was annotated using ANNOVAR [56]. Prioritizations of variants found in the whole exome data are shown in Fig. 2. The number of variants after every filtering step is given in Supplementary Table S3. From the annotated variants: the first filtering step (F1) variants that are non-synonymous and exonic were selected, the second filter (F2) selected variants that have allele frequency ≤ 0.05, and the third filtering step (F3) selected variants that are predicted as deleterious by any two of the predicting software (SIFT, PolyPhen2 or Mutation Taster) [60–62] for further analysis. Frequently mutated genes which are reported in leukemia patients were listed out after performing data mining through literature survey as well as which are catalogued in databases (Supplementary Table S4). F2 and F3 were then matched with the list of frequently mutated genes in leukemia (F4). The observed variants were interpreted using CIViC [63] and ClinVar database [64], while variants not present in CIViC and ClinVar were interpreted using dbSNP [65] and OMIM database [66]. The allele frequency was also compared using databases like ExAc [67], gnoMAD [68], ESP6500 (https://evs.gs.washington.edu/EVS/), 1000genomes [69], IndiGenomes [53] and SAGE [54].

## Supplementary Information

**Additional file 1.**

**Additional file 2.**

## Data Availability

Alignment files (.bam) that support the findings of this study have been deposited in SRA with the accession codes PRJNA774922.

## Abbreviations

ALL: Acute Lymphoblastic Leukemia
AML: Acute Myeloid Leukemia
ASXL: ASXL Transcriptional Regulator 1
BCL10: BCL10 immune signalling adaptor
CIViC: Clinical Interpretation of Variants in Cancer
CML: Chronic Myeloid Leukemia
Erk: Extracellular Signal Regulated Kinase
ExAc: Exome Aggregation Consortium
FLT3-ITD: Fms Related Receptor Tyrosine Kinase 3
GATK: Genome Analysis Toolkit
HGMD: Human Gene Mutation Database
JCML: Juvenile Chronic Myelogenous Leukemia
MAP: MUTYH Associated Polyposis
MLL: Myeloid Lymphoid Leukemia
MUTYH: MutY DNA Glycosylase
NGS: Next Generation Sequencing
NOTCH1: Neurogenic locus notch homolog protein 1
OMIM: Online Mendelian Inheritance in Man
PEST: Proline (P), glutamic acid (E), serine (S), and threonine (T)
PTP: Protein Tyrosine Phosphatase
PTPN11: Protein Tyrosine Phosphatase Non-Receptor Type 11
QC: Quality Control
SAGE: South Asian Genomes and Exomes
SIFT: Sorting Intolerant From Tolerant
STAT: Signal Transducer and Activator of Transcription proteins
VCF: Variant Calling File
WES: Whole Exome Sequencing
WT-FLT3: Wildtype-Fms Related Receptor Tyrosine Kinase 3
