# Supplementary material for: Whole exome sequencing of pediatric leukemia reveals a novel InDel within FLT-3 gene in AML patient from Mizo tribal population, Northeast India
Source: BMC Genom Data. 2022 Mar 28;23:23. doi: 10.1186/s12863-022-01037-x (PMC8961913; doi:10.1186/s12863-022-01037-x)
Supplement: Supplementary file 1 — Additional file 1. [file 12863_2022_1037_MOESM1_ESM.docx]

**Supplementary Figures**

**Supplementary Figure 1:** Point mutation showing G>A change in *MUTYH.*


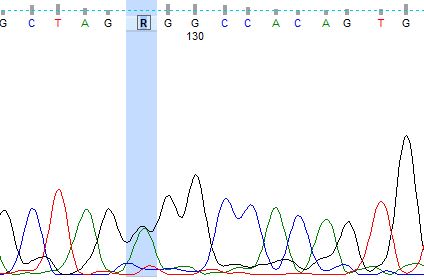


Heterozygous mutation leads to a peak under peak upon Sanger sequencing of *MUTYH* where it denotes as “R”.

**Supplementary Figure 2:** Point mutation showing T>A change in *NOTCH1.*


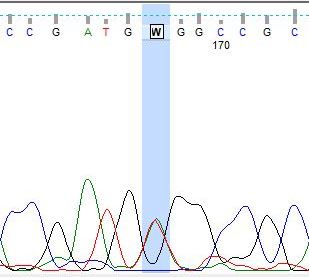


Heterozygous mutation leads to a peak under peak upon Sanger sequencing if *NOTCH1* where it denotes as “R”.

**Supplementary Figure 3:** Heterozygous T>C change in PTPN11.


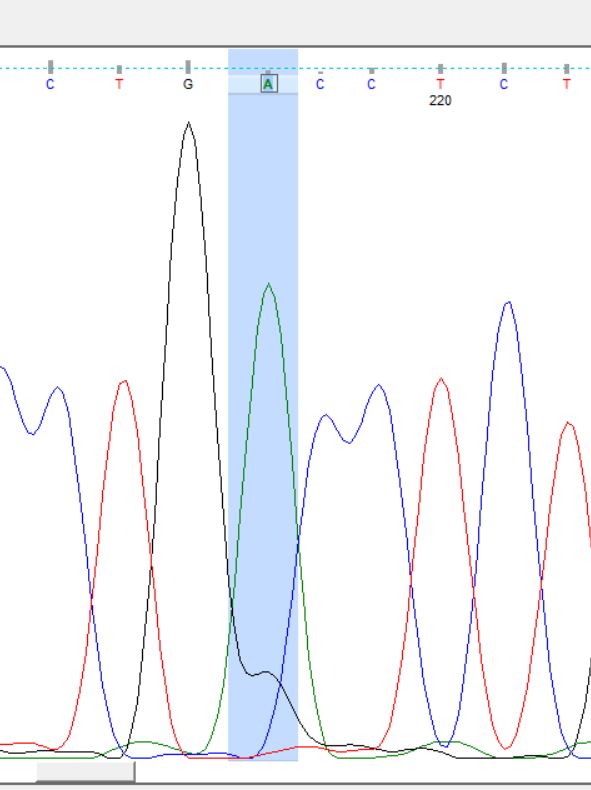


Using reverse primer, T>C change is observed as depicted by a slight peak as well as an irregularity in the spacing of the bases (between G and A) in the called base is observed.

**Supplementary Figure 4:** Evidence from NGS data showing Novel Indel in *FLT3* in exon 14


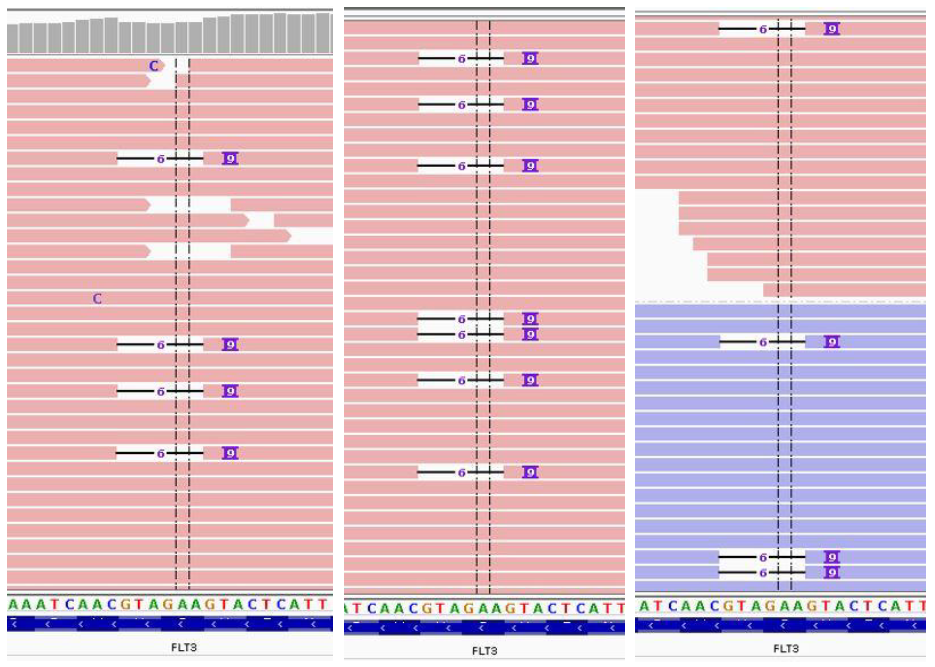


BAM file of Novel Indel in FLT3 was viewed using IGV that show the sequencing reads of the deleted and inserted region within Exon 14.

**Supplementary Figure 5:** Novel Indel in *FLT3* in exon 14*.*


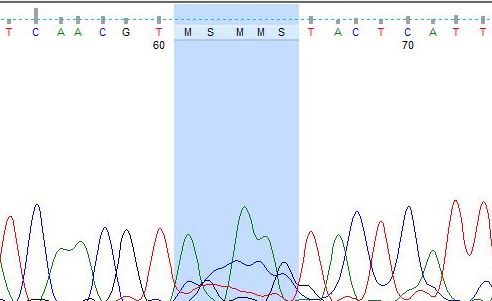


Sanger sequencing reveals Novel Indel region in the juxtamembrane domain of *FLT3*
