# Supplementary material for: Whole exome sequencing of pediatric leukemia reveals a novel InDel within FLT-3 gene in AML patient from Mizo tribal population, Northeast India
Source: BMC Genom Data. 2022 Mar 28;23:23. doi: 10.1186/s12863-022-01037-x (PMC8961913; doi:10.1186/s12863-022-01037-x)
Supplement: Supplementary file 2 — Additional file 2. [file 12863_2022_1037_MOESM2_ESM.docx]

**Supplementary Table S1:** List of samples collected and their Clinical conditions

| **Sample ID** | **Age (years)** | **Gender** | **Clinical Condition** |
| --- | --- | --- | --- |
| GDN4252 | 13 | F | ALL |
| GDN4253 | 11 | M | ALL |
| GDN4254  GDN4263 | 14  38 | M  F | ALL  Mother |
| GDN4255^*^ GDN4264 | 16  42 | M  **F** | AML – M1  Mother |
| GDN4256 | 12 | M | ALL |
| GDN4257 GDN4265 | 11  32 | M  **F** | AML  Mother |
| GDN4258^**^ GDN4266 | 2  36 | F  F | AML – M1  Mother |
| GDN4259 | 5 | M | JCML |
| GDN4260 | 4 | M | ALL |
| GDN4261^***^ | 2 | F | AML - M1 |
| GDN4262 | 4 | M | CML |

^*^NOTCH1 mutation was observed. ^**^MUTYH mutation was observed. ^***^FLT3 and PTPN11 mutations were observed.

**Supplementary Table S2**: List of sequencing reads that passed quality check and aligning to the reference genome with the percentage for each leukemic patients. QC: Quality control, %; Percentage.

| **Samples** | **QC pass reads** | **Aligned reads** | **% of alignment** |
| --- | --- | --- | --- |
| GDN4252 | 64265254 | 64261094 | 99.99 |
| GDN4253 | 35548941 | 35546525 | 99.99 |
| GDN4254 | 84911078 | 84907234 | 99.99 |
| GDN4255 | 75146989 | 75143138 | 99.9 |
| GDN4256 | 30669406 | 30658577 | 99.96 |
| GDN4257 | 10760797 | 10755716 | 99.95 |
| GDN4258 | 33683692 | 33668629 | 99.96 |
| GDN4259 | 64428370 | 64404387 | 99.96 |
| GDN4260 | 49560787 | 49557628 | 99.99 |
| GDN4261 | 59679153 | 59675442 | 99.99 |
| GDN4262 | 65613087 | 65608945 | 99.99 |
| **MEAN** | **52206141** | **52198847** | **99.97** |

**Supplementary Table S3**: List of frequently mutated genes in leukemia.

| NRAS | NF1 | CBLB | CRLF2 | CEBPA | IDH2 |
| --- | --- | --- | --- | --- | --- |
| KRAS | NPM1 | KMT2A | SETBP1 | PDGFRB | CTCF |
| FLT3 | BCR | KMT2D | BCL2L11 | U2AF1 | PTPN11 |
| ABL1 | JAK2 | CREBBP | CSF1R | PDGFRA | GATA2 |
| ATM | BCL10 | EP300 | BTK | IKZF1 | CDNK2A |
| KIT | BIRC3 | ARID1A | GNAS | MTOR |  |
| JAK3 | TP53 | ASXL1 | MYD88 | NT5C2 |  |
| CSF3R | JAK1 | TET2 | BIRC3 | DNMT3A |  |
| RUNX1 | NPM1 | PAX5 | POT1 | IKZF3 |  |
| ETV6 | NOTCH1 | IKZF2 | IDH1 | WT1 |  |

**Supplementary Table S4**: List of total number of variants in each sample from the raw annotated files, after the different filters (filter1, filter2 and filter3)

| **Samples** | **Annotated file** | **After Filter1** | **After filter 2** | **After filter 3** |
| --- | --- | --- | --- | --- |
| GDN4252 | 512926 | 12980 | 1699 | 129 |
| GDN4253 | 348029 | 12476 | 1732 | 148 |
| GDN4254 | 683227 | 13022 | 1884 | 162 |
| GDN4255 | 743429 | 12798 | 1823 | 147 |
| GDN4256 | 337397 | 12723 | 1902 | 154 |
| GDN4257 | 129832 | 11783 | 2120 | 234 |
| GDN4258 | 331558 | 12754 | 1865 | 168 |
| GDN4259 | 557585 | 12808 | 1739 | 136 |
| GDN4260 | 534159 | 12811 | 1848 | 165 |
| GDN4261 | 668326 | 12914 | 1767 | 160 |
| GDN4262 | 656041 | 13015 | 1850 | 144 |
| **Mean** | **500228** | **12735** | **1839** | **159** |

**Supplementary Table S5**: List of Non-synonymous variants that matched with variants from ClinVar with their Clinical Significance and the Disease associated in ClinVar

| **Chr** | **Pos** | **Ref** | **Alt** | **Gene** | **Clinical Significance from ClinVar** | **Disease associated** |
| --- | --- | --- | --- | --- | --- | --- |
| 11 | 108098555 | A | G | ATM | Conflicting interpretations of Pathogenicity | Ataxia-telangiectasia syndrome, Hereditary cancer-predisposing syndrome |
| 11 | 108159732 | C | T | ATM | Benign / Likely Benign | Ataxia-telangiectasia syndrome, Hereditary cancer-predisposing syndrome |
| 11 | 119156193 | C | T | CBL | Benign / Likely Benign | Rasopathy, Noonan-Like Syndrome Disorder |
| 12 | 49434409 | G | A | KMT2D | Benign | Kabuki syndrome |
| 1 | 45797401 | G | A | MUTYH | Conflicting interpretations of Pathogenicity | MYH-associated polypopsis, Hereditary cancer-predisposing syndrome |
| **1** | **45797914** | **C** | **T** | **MUTYH** | **Pathogenic / Likely Pathogenic** | **MYH-associated polypopsis, Hereditary cancer-predisposing syndrome** |
| 1 | 45800146 | C | T | MUTYH | Benign, Uncertain Significance | MYH-associated polypopsis, Hereditary cancer-predisposing syndrome |
| 1 | 45800167 | G | A | MUTYH | Benign, Uncertain Significance | MYH-associated polypopsis, Hereditary cancer-predisposing syndrome |
| 18 | 42643270 | G | T | SETBP1 | likely Benign | Schinzel-Giedion syndrome |
| 1 | 85742023 | C | A | BCL10 | Benign | Immunodeficiency 37 |
| 20 | 31022469 | G | A | ASXL1 | Benign | C-like syndrome |
| 22 | 23654017 | G | A | BCR | Uncertain Significance | ALL and AML |
| 4 | 106158550 | G | T | TET2 | Not provided |  |
| 4 | 55589830 | A | G | KIT | Uncertain Significance | Gastrointestinal stroma tumor |
| 9 | 139401375 | C | T | NOTCH1 | Uncertain Significance | Adams-Oliver syndrome 5, Cardiovascular phenotype |
| 9 | 139410139 | T | C | NOTCH1 | Uncertain Significance | Adams-Oliver syndrome 5 |
